# A New Way of Investigating the Relationship Between Fasting Blood Sugar Level and Drinking Glucose Solution

**DOI:** 10.3389/fnut.2022.862071

**Published:** 2022-05-10

**Authors:** Muhammad Aslam, Mohammed Albassam

**Affiliations:** Department of Statistics, Faculty of Science, King Abdulaziz University, Jeddah, Saudi Arabia

**Keywords:** classical statistics, imprecise observations, medical data, neutrosophy, simulation

## Abstract

The existing *t*-test of a correlation coefficient works under a determinate environment. In uncertainty, the existing *t*-test of a correlation coefficient is unable to investigate the significance of correlation. The study presents a modification of the existing *t*-test of a correlation coefficient using neutrosophic statistics. The test statistic is designed to investigate the significance of correlation when imprecise observations or uncertainties in the level of significance are presented. The test is applied to data obtained from patients with diabetes. From the data analysis, the proposed *t*-test of a correlation coefficient is found to be more effective than existing tests.

## Introduction

Correlation analysis is conducted to see the degree of relationship between two variables. Correlation analysis helps in determining the positive or negative correlation between two variables. The value of a correlation coefficient lies between −1 and +1. The computed value of the correlation coefficient from data always lies in this interval. Statistical tests have been conducted in various fields for decision-making purposes. Among the statistical tests, the *t*-test for correlation is applied to investigate the significance of the correlation between two variables. In the *t*-test for correlation, the null hypothesis that there is no association between two variables is tested against the alternative hypothesis that two variables are associated. Values of the statistic of the *t*-test for correlation are calculated from given data and compared with tabulated values. The null hypothesis of no association between two variables is accepted if the calculated value is less than the tabulated value. Bartroff and Song (1) conducted a correlation analysis to investigate the relationship between impact factors and the ranking of electrical journals. Aleixandre-Benavent et al. (2) discussed the correlation between impact factors and published papers' research data. McGillivray and Astell (3) provided a correlation between usage and citations of open access journals. For more information, the reader may refer to Tang and Landes (4) and e Silva et al. (5).

Correlation analysis has been widely applied in medical research. It is conducted to investigate the association between variables causing a specific disease. Schober et al. (6) applied correlation analysis on anesthesia data. Najmi and Balakrishnan Sadasivam (7) provided guidelines for medical students related to statistical tests. Statistical analysis has also been conducted to investigate the relationship among various causes of diabetes.Khan et al. (8) discussed a statistical analysis for patients with diabetes. Liu et al. (9) applied various statistical methods for data on diabetes. Wani et al. (10) investigated the effect of weight and smoking on type-2 diabetes. Nedyalkova et al. (11) presented a statistical analysis on type-2 diabetes. Adaobi et al. (12) presented an analysis using blood glucose data. More information can be seen in Mukasheva et al. (13), Eynizadeh et al. (14), Balamurugan et al. (15), Alsaqr (16), Janse et al. (17), and Sun et al. (18).

Neutrosophic logic was introduced by Smarandache (19), which is the generalization of fuzzy-logic. The former gives information about three measures (truth, false, and indeterminacy), while the latter gives information about two measures (truth and false). Smarandache (20) showed the efficiency of neutrosophic logic over fuzzy-logic and interval-based analysis. Neutrosophic logic has many applications in medical science. Ansari et al. (21) discussed an application of neutrosophic sets in artificial intelligence. Jafar et al. (22) used the neutrosophic logic in medical diagnosis. Basha et al. (23) applied neutrosophic logic in the classification of X-rays of the chest of patients with coronavirus disease 2019 (COVID-19). More information on applications of neutrosophic logic in medical science can be seen in Zhang et al. (24) and Zhang et al. (25). Neutrosophic statistics was developed by Smarandache (26) using the idea of neutrosophy in numbers. Chen et al. (27) and Chen et al. (28) discussed methods to analyze neutrosophic data. Aslam et al. (29) applied neutrosophic statistics on diabetics' data. Ling et al. (30) analyzed neutrosophic numbers in medical waste treatment. More applications can be seen in Das et al.'s studies (31) and Saeed et al.'s studies (32).

Aslam (33) proposed a neutrosophic Z-test for two samples to investigate the relationship between metrological variables. By exploring the literature and to the best of our knowledge, there is still a gap to work on *t*-test for correlation under neutrosophic statistics for a single sample. We will present the design of a *t*-test for correlation using neutrosophic statistics in this study. The neutrosophic statistic will be given and applied using data obtained from patients with diabetes. We expect that the proposed *t*-test for correlation will beat the existing *t-*tests for correlations in terms of information, adequacy, and flexibility.

## Method

The existing *t*-test of a correlation coefficient using classical statistics works only when decision-makers are sure about the parameters involved in the implementation of the test. In practice, it may not be possible to the level of significance, sample size, and observations obtained from a measurement or a complex process are always certain; see, for example, Doll and Carney (34). Modification of the existing *t*-test of a correlation coefficient is needed to investigate the significance of the correlation between variables in an indeterminate environment. Now, we will develop a *t*-test of a correlation coefficient using neutrosophic statistics with the expectation that the proposed test will be efficient and a general form of the existing *t*-test of a correlation coefficient. The procedure of the proposed *t*-test of a correlation coefficient using neutrosophic statistics will be explained as: Let *X*_*N*_ = *X*_*L*_+*X*_*U*_*I*_*X*_*N*__; *I*_*X*_*N*__ϵ[*I*_*X*_*L*__, *I*_*X*_*U*__] and *Y*_*N*_ = *Y*_*L*_+*Y*_*U*_*I*_*Y*_*N*__; *I*_*Y*_*N*__ϵ[*I*_*Y*_*L*__, *I*_*Y*_*U*__] be neutrosophic random variables, where the first values denote the determinate parts, the second values denote the indeterminate parts, and *I*_*X*_*N*__ϵ[*I*_*X*_*L*__, *I*_*X*_*U*__], *and I*_*Y*_*N*__ϵ[*I*_*Y*_*L*__, *I*_*Y*_*U*__] are indeterminacy. Let *n*_*N*_ = *n*_*L*_+*n*_*U*_*I*_*n*_*N*__; *I*_*n*_*N*__ϵ[*I*_*n*_*L*__, *I*_*n*_*U*__] be a neutrosophic random sample of size *n*_*N*_ϵ[*n*_*L*_, *n*_*U*_], and α_*N*_ = α_*L*_+α_*U*_*I*_α*N*_; *I*_α*N*_ϵ[*I*_α*L*_, *I*_α*U*_] be a level of significance under uncertainty, where *n*_*L*_ and, α_*L*_ are determinate values, *n*_*U*_*I*_*n*_*N*__ *and α*_*U*_, *I*_α*N*_ are indeterminate values, and *I*_*n*_*N*__ϵ[*I*_*n*_*L*__, *I*_*n*_*U*__] and *I*_α*N*_ϵ[*I*_α*L*_, *I*_α*U*_] are measures of uncertainty. Suppose that (*X*_*iN*_, *Y*_*iN*_) to be pair data and let *r*_*N*_ = *r*_*L*_+*r*_*U*_*I*_*r*_*N*__; *I*_*r*_*N*__ϵ[*I*_*r*_*L*__, *I*_*r*_*U*__] be a neutrosophic correlation, where *r*_*L*_ is a determinate part, the *r*_*U*_*I*_*r*_*N*__ is an indeterminate part, and *I*_*r*_*N*__ϵ[*I*_*r*_*L*__, *I*_*r*_*U*__] is the measure of correlations. The neutrosophic correlation *r*_*N*_ϵ[*r*_*L*_, *r*_*U*_], by following Aslam and Albassam (35), is defined as:


(1) $$\begin{array}{l} \begin{array}{c} r_{N}=\frac{\sum_{i=1}^{n_{L}}\left(X_{iL}-{\bar{X}}_{L})(Y_{iL}-{\bar{Y}}_{L}\right)}{\sqrt{\sum_{i=1}^{n_{L}}{\left(X_{iL}-{\bar{X}}_{L}\right)}^{2}\sum_{i=1}^{n_{L}}{\left(Y_{iL}-{\bar{Y}}_{L}\right)}^{2}}}+\\ \frac{\sum_{i=1}^{n_{U}}\left(X_{iU}-{\bar{X}}_{U})(Y_{iU}-{\bar{Y}}_{U}\right)}{\sqrt{\sum_{i=1}^{n_{U}}{\left(X_{iU}-{\bar{X}}_{U}\right)}^{2}\sum_{i=1}^{n_{U}}{\left(Y_{iU}-{\bar{Y}}_{U}\right)}^{2}}}I_{r_{N}}; \end{array}\\ \begin{array}{c} I_{r_{N}}\epsilon [I_{r_{L}},I_{r_{U}}] \end{array} \end{array}$$

where *r*_*L*_ = *r*_*U*_, and the neutrosophic correlation *r*_*SN*_ϵ[*r*_*L*_, *r*_*U*_] can be written as:


(2) $$\begin{array}{l} \begin{array}{c} r_{SN}=(1+I_{r_{SN}})\frac{\sum_{i=1}^{n_{S}}\left(X_{iS}-{\bar{X}}_{S})(Y_{iS}-{\bar{Y}}_{S}\right)}{\sqrt{\sum_{i=1}^{n_{S}}{\left(X_{iS}-{\bar{X}}_{S}\right)}^{2}\sum_{i=1}^{n_{S}}{\left(Y_{iS}-{\bar{Y}}_{S}\right)}^{2}}}; \end{array}\\ \begin{array}{c} I_{r_{SN}}\epsilon [I_{r_{SL}},I_{r_{SU}}] \end{array} \end{array}$$

Note that the neutrosophic correlation *r*_*N*_ϵ[*r*_*L*_, *r*_*U*_] is a generalization of the existing correlation under classical statistics. The neutrosophic correlation *r*_*N*_ϵ[*r*_*L*_, *r*_*U*_] reduces to correlation using classical statistics when *I*_*r*_*SL*__= 0. To test the null hypothesis *H*_0*N*_ that there is no correlation between the variables vs. the alternative hypothesis *H*_1*N*_ that both variables are associated, the neutrosophic test statistic *t*_*N*_ϵ[*t*_*L*_, *t*_*U*_] is defined as:


(3) $$\begin{array}{r} t_{N}=t_{L}+t_{U}I_{t_{N}}\epsilon \left[I_{t_{L}},I_{t_{U}}\right] \end{array}$$

The alternative form of *t*_*N*_ϵ[*t*_*L*_, *t*_*U*_] is defined as:


(4) $$\begin{array}{l} \begin{array}{c} t_{N}=\frac{r_{L}}{\sqrt{1-r_{L}^{2}}}\times \sqrt{n_{L}-2}+\frac{r_{U}}{\sqrt{1-r_{U}^{2}}}\times \end{array}\\ \begin{array}{c} \sqrt{n_{U}-2}I_{t_{N}};I_{t_{N}}\epsilon [I_{t_{L}},I_{t_{U}}] \end{array} \end{array}$$

where *t*_*L*_ = *t*_*U*_ and the neutrosophic correlation *t*_*SN*_ ϵ [*t*_*SL*_, *t*_*SU*_] can be written as:


(5) $$\begin{array}{r} t_{SN}=\left(1+I_{t_{SN}}\right)\frac{r_{SN}}{\sqrt{1-r_{SN}^{2}}}\times \sqrt{n_{S}-2};I_{t_{SN}}\epsilon \left[I_{t_{SL}},I_{t_{SU}}\right] \end{array}$$

Note that *t*_*N*_ϵ[*t*_*L*_, *t*_*U*_] follows the neutrosophic *t*-distribution with the degree of freedom *n*_*N*_−2.

Note that the neutrosophic statistics *t*_*N*_ϵ[*t*_*L*_, *t*_*U*_] is a generalization of the existing statistics under classical statistics. The neutrosophic statistics *t*_*N*_ϵ[*t*_*L*_, *t*_*U*_] reduces to statistic using classical statistics when *I*_*r*_*SL*__= 0.

The proposed *t*-test of a correlation coefficient will be carried out through the following steps:

Step-1: state *H*_0*N*_:*r*_*N*_ϵ[*r*_*L*_, *r*_*U*_] = 0 vs. *H*_1*N*_:*r*_*N*_ϵ[*r*_*L*_, *r*_*U*_]≠ 0;.
Step 2: fix the level of significance α_*N*_ = α_*L*_+α_*U*_*I*_α*N*_; *I*_α*N*_ϵ[*I*_α*L*_, *I*_α*U*_] and select the tabulated value *t*_*C*_ from (36);
Step 3: compute statistic *t*_*N*_ϵ[*t*_*L*_, *t*_*U*_] and compare with *t*_*C*_;.
Step 4: do not reject *H*_0*N*_:*r*_*N*_ϵ[*r*_*L*_, *r*_*U*_] = 0 if*t*_*N*_ϵ[*t*_*L*_, *t*_*U*_] < *t*_*C*_.

## Application Using Data On Diabetes

**Figure 1:**
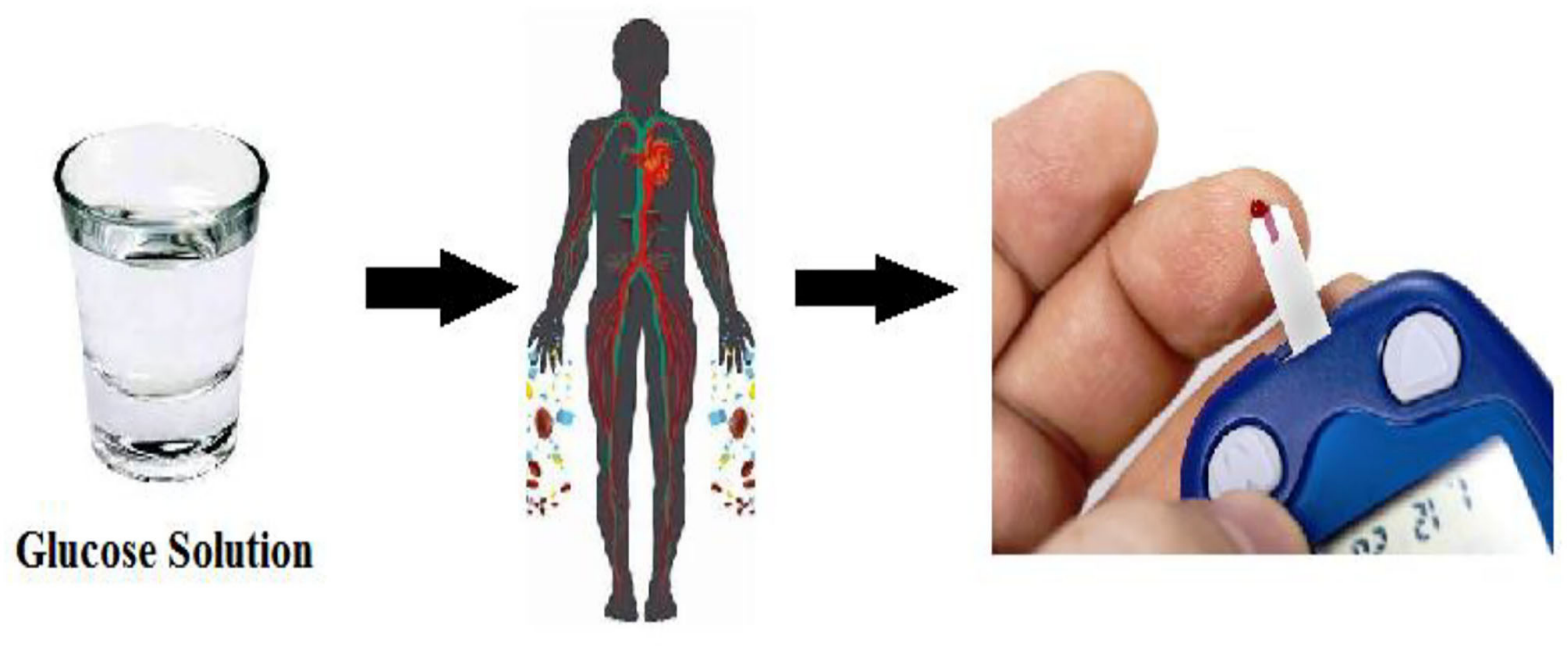
Schematic diagram of the data measurement of sugar level in blood.

To investigate the significance of the correlation between the sugar level and Drinking Glucose Solution about 237 ml contained 75 g of sugar, the data from 320 diabetics patients aged from 45 to 60 were collected from a hospital located in Hafizabad, Pakistan. A group of 20 patients at each age level is formed and the minimum and maximum blood sugar levels are recorded from 16 groups of patients. The schematic diagram to measure blood sugar level is depicted in Figure 1.

The data of blood sugar levels are reported in Table 1. The minimum and maximum levels of blood sugar (in mg/dl) after 8 h of fasting (G1) and 2 h after drinking, the glucose solution of about 237 ml and containing 75 g sugar (G2) are shown in Table 1. From Table 1, it can be seen that blood sugar level is expressed in intervals; therefore, investigation on the significance of correlation cannot be performed using the existing *t*-test for correlation. Decision-makers can apply the proposed *t*-test for correlation using neutrosophic statistics. To test the null hypothesis *H*_0*N*_ that there is no correlation between G1 and G2 vs. *H*_1*N*_ that G1 and G2 are associated, the neutrosophic correlation *r*_*N*_ϵ[*r*_*L*_, *r*_*U*_] for G1 and G2 is calculated as follows: *r*_*N*_ = 0.9900−0.9899*I*_*r*_*N*__; *I*_*r*_*N*__ϵ[0, 0.0001]. The value of statistic *t*_*N*_ϵ[*t*_*L*_, *t*_*U*_] for G1 and G2 is calculated as: *t*_*N*_ = 26.29−26.22*I*_*t*_*N*__ϵ[0, 0.0027].

**Table 1:** Data of sugar levels in the blood.

**Age (years)**	**Data of “G1” (mg/dL)**	**Data of “G2” (mg/dL)**
45	[159, 199]	[166, 206]
46	[150, 196]	[156, 202]
47	[139, 199]	[147, 207]
48	[142, 167]	[148, 173]
49	[152, 210]	[160, 218]
50	[143, 187]	[150, 194]
51	[151, 177]	[159, 185]
52	[140, 195]	[147, 207]
53	[154, 200]	[160, 206]
54	[142, 197]	[149, 204]
55	[150, 189]	[157, 196]
56	[160, 198]	[168, 206]
57	[162, 190]	[170, 198]
58	[146, 198]	[152, 204]
59	[149, 188]	[155, 194]
60	[177, 198]	[179, 205]

To investigate the relationship between G1 and G2, the following steps will be carried out:

Step 1: state *H*_0*N*_: no correlation between G1 and G2 vs. *H*_1*N*_: G1 and G2 are associated;
Step 2: fix the level of significance α_*N*_ = 0.05 and the tabulated value is *t*_*C*_= 1.76 at the degree of freedom 14 from (36);
Step 3: compute statistic *t*_*N*_ϵ[*t*_*L*_, *t*_*U*_] = [26.29, 26.22] and compare with *t*_*C*_ = 1.76;
Step 4: As [26.29, 26.22 > 1.76], it is concluded that blood sugar levels between G1 and G2 are significant.

Based on the analysis, it can be concluded that there is a significant correlation between 8-h fasting sugar level and 2 h after drinking, the glucose solution of about 237 ml and containing75 g of sugar.

## Advantages

The proposed *t*-test for correlation is a generalization of *t*-test for correlation using classical statistics, interval-based statistics, and fuzzy logic. Now, the efficiency of the proposed *t*-test for correlation will be discussed over these tests in terms of flexibility and information. For comparisons, we will consider the neutrosophic form of the statistic *t*_*N*_ϵ[*t*_*L*_, *t*_*U*_] that is *t*_*N*_ = 26.29−26.22*I*_*t*_*N*__ϵ[0, 0.0027]. This neutrosophic form has two parts of information: the first one is about the statistic of classical statistics, and the second one is about the indeterminate part of the proposed test. For example, when *t*_*L*_= 0, the value 26.29 presents the value of test statistic using classical statistics. According to the proposed test, the value of *t*_*N*_ϵ[*t*_*L*_, *t*_*U*_] will in the interval from 26.29 to 26.22 rather than the exact value. The proposed test also indicates the measure of indeterminacy associated with the interval that is 0.0027. From this comparison, it is clear that the *t*-test using neutrosophic statistics has an edge over the existing *t*-test for correlation. The *t*-test using interval statistics and fuzzy-based logic gives the values of the test statistic in intervals without giving any information about the measure of indeterminacy. For example, for testing the hypothesis *H*_0*N*_: no correlation between G1 and G2, the probability of committing a type-1 error is 0.05 (false), the probability of accepting *H*_0*N*_: no correlation between G1 and G2 is 0.95 (true), and the measure of indeterminacy is 0.0027. The *t*-test using fuzzy logic will give information only about the measures of falseness and truth. Based on the analysis, it is concluded that the proposed *t*-test for correlation is better than the existing tests.

## Discussions

As the data is collected from a group having 20 people at the fasting time and then after two hours of drinking glucose solution about 237 milliliters contained 75-gram. The neutrosophic form of the correlation between G1 and G2 is *r*_*N*_ = 0.99−0.9899*I*_*r*_*N*__; *I*_*r*_*N*__ϵ[0, 0.0001]. It is interesting to note that the correlation between the two groups, G1 and G2, varies from 0.99 to 0.9899, with the measure of indeterminacy *I*_*r*_*N*__= 0.001. From this correlation analysis, it can be seen that there is a strong positive correlation between fasting of 8 h and after 2 h of drinking the glucose solution. It means that if an 8-h fasting blood sugar level is high, then the blood sugar level after 2 h of drinking the glucose solution is also high and vice versa. It is important to note that after the 8-h fasting, the minimum blood sugar level of those aged 45 is 159. The value 159 indicates that these patients should take some energy drink before 2 h before sleeping, so that blood sugar can be utilized properly by the body. In addition, with an increase in 8-h fasting sugar, patients aged 45 to 60 should avoid taking carbohydrate or glucose items.

## Concluding Remarks

The *t*-test of a correlation coefficient under neutrosophic statistics was presented in the article. The proposed *t*-test of a correlation coefficient was a generalization of the existing *t*-test of a correlation coefficient under classical statistics. From the real example, the proposed *t*-test of a correlation coefficient was found to be effective for investigating the significance of correlation in an indeterminate environment. The simulation study showed that measures of indeterminacy affect the decision on the significance of correlation. The proposed test can be applied to investigate correlations in the fields of economics, business, medicine, and industry. The proposed *t*-test of a correlation coefficient using a double sampling scheme can be considered as future research. Further statistical properties can be studied in future research. The proposed study can be extended for blood sugar measurement under different conditions and validation methods as future research. In addition, some disturbances can also be considered for blood glucose measurement in future studies.

## Data Availability Statement

The original contributions presented in the study are included in the article/supplementary material, further inquiries can be directed to the corresponding author/s.

## Author Contributions

Both authors listed have made a substantial, direct, and intellectual contribution to the work and approved it for publication.

## Funding

This work was funded by the Deanship of Scientific Research at King Abdulaziz Univesity.

## Conflict of Interest

The authors declare that the research was conducted in the absence of any commercial or financial relationships that could be construed as a potential conflict of interest.

## Publisher's Note

All claims expressed in this article are solely those of the authors and do not necessarily represent those of their affiliated organizations, or those of the publisher, the editors and the reviewers. Any product that may be evaluated in this article, or claim that may be made by its manufacturer, is not guaranteed or endorsed by the publisher.
